# BioBanking - The Holy Grail of novel drug and diagnostic developments?

**DOI:** 10.1186/2043-9113-1-14

**Published:** 2011-05-13

**Authors:** György Marko-Varga

**Affiliations:** 1 Div. Clinical Protein Science & Imaging, Dept. of Measurement Technology and Industrial Electrical Engineering, Lund University Biomedical Center, BMC C13 SE-221 84 Lund, Sweden; 2 First Department of Surgery, Tokyo Medical University, 6-7-1 Nishishinjiku Shinjiku-ku, Tokyo, 160-0023 Japan

## Abstract

The ever increasing social cost that society pays for illness and disease are currently steadily increasing in many countries in the world today. These changes in society becomes a major financial burden that activates politicians and health care organizations in order to find new solutions. Biobanks are becoming the new powerful modality within the field of modern Life Science, that is expected to be important in the proactive awareness of patient health status. Biobanks are also expected to promote the developments of targeted treatments with personalized indicator assays, for effective use of Personalized Medicine treatments in the near future.

## 

The reason for these effects of growing health care costs we are experiencing springs from several changes and developments, such as increased patient demands, patient awareness of quality of life, and an increasing global population. Last but not least, the increasing number of elderly people, and the health care implications for future technology developments, are challenges that we need to address in order to be able to be pro-active and build a future plan that meets the resources that will become evident with time. These future developments were recently highlighted in a"White Paper" by representatives from Pharma industry, academic research institutes, companies that are technology platform providers and medical health institutions in Japan, a country with an expected elderly population of 40% in 2050, [1].

We have reached a point where the bioinformatic developments have given us the basis for taking on the sequencing of the "Human Proteome", that is coded by the genome, ever since the last decade when the Human Genome Project was announced by Bill Clinton and Tony Blair, as a scientific milestone. The gene centric Human Proteome Project (HPP) approach was recently initiated by HUPO, at the 9th World Congress in Sydney, 2010, where research teams established chromosome consortia. The start of this HPP chromosome initiative will need major effort and builds within the UniProt database, as well as establishment of novel informatic tools that can aid in delivering on the sequence content of the Human Proteome. Given this upcoming scientific milestone challenge ahead of us, the logical question at hand is: What is the best way forward in getting clinical and disease understanding with the gene coded protein sequences at hand?

One important opportunity could be the current activities on building Biobank archives. Currently, there are major interests and dedicated projects with substantial resources to build biobanking facilities around the world. Biobanks hold archives of population, and disease-based patient samples that offer a highly valuable sample collection that could form the future knowledge base to the scientific community. Clinical registers that describe the sample history and the diagnosis are of high value, that strive to correlate biological changes in disease to changes on a molecular level.

Clearly there are the expectations from politicians and health care leadership, that by building large collections of highly standardized and well characterized patient samples, there will be a future scientific solution to many of the challenges we have by treating patients today. In order to be able to build these Biobanks with high quality standards, as well as the procedures whereby access to these treasures needs to include the protection of patient integrity. Clearly, ethical standards and data management procedures are central when embarking into this new scale of sample and data processing endeavor.

No doubt there are major Biobank stakeholders in this new field, where major investments are currently being made, that are awaiting novel solutions of future Biomarker deliverables, such as preventive and drug-targeted biomarkers, as well as new imaging diagnostic technologies. These new conceptual developments are especially urgent due to a high unmet need within diseases such as cancers, obesity, diabetes, cardiovascular diseases, and neurodegenerative diseases. Introducing the Biobank concept as a new powerful modality within the field of modern Life Science, is expected to be important in pro-active awareness of patient health status. The pro-active concept should be seen as the future for many countries, instead of the act-on-demand procedure, where the awareness of treatment occurs when the patient already has serious disease advancement. Most countries act on patient disease onset where the costs and prevention of disease are found to be less optimal, than in countries where pro-active health-status check up is the standard procedure. Biobanks are also expected to promote the developments of targeted treatments with personalized indicator assays, for effective use of Personalized Medicine treatments in the near future[2].

Large biobank facilities equipped with robotics and automated sample processing will also become a large asset to drug companies. As the pharmaceutical industry has passed the period of poor candidate drug generation, the filled up pipelines of drug compounds can now readily be processed through a validation process utilizing selected and dedicated clinical material from Bio-Sample repositories around the world. In addition, each biofluid and/or tissue sample will most probably have associated clinical data, from where the patient cohorts can be composed. It is also envisioned that the Biobanking initiatives will generate a whole new dimension of data sets from expression studies. This new data will be a valuable delivery, and payback for accessing the treasures within Biobanks. It is with great interest that we will follow the maturation of the mechanistic disease pathophysiology, where the HUPO Chromosome Consortia in collaborative efforts with the Proteomics society will build the future basis of the human proteome. All of the delivered outcomes will be publicly available in UniProt [3].

Another objective that needs to be met, will be the protein data integration, with functional networks that will provide us with a comprehensive data-set, to be used as a public resource. How big role the Biobanks will play in all of these scientific milestone developments, and whether this will become the Holy Grail, and whether the benefit by preventive and participatory medicine for patients combined with stratified diagnostics developments - that still remains to be proven by all of us in the scientific, and industrial community.

## List of abbreviations

HPP: Human Proteome Project

## Competing interests

The author declares that they have no competing interests.

## Author information

György Marko-Varga

Dr Marko-Varga has a major research focus within the clinical field where pathophysiological mechanisms in disease evolvement of proteins in lung cancer, COPD and cardiovascular diseases are investigated. Drug characterization is another research area of Dr Marko-Varga, with the establishment of biobanks and imaging resources in collaboration with industry and clinical hospitals in Europe, US and Asia.
